# Methods of liberation from mechanical ventilation: Which one is best?

**DOI:** 10.3389/fmed.2022.917369

**Published:** 2022-08-16

**Authors:** Ling Liu

**Affiliations:** Jiangsu Provincial Key Laboratory of Critical Care Medicine, Department of Critical Care Medicine, School of Medicine, Zhongda Hospital, Southeast University, Nanjing, China

**Keywords:** weaning from mechanical ventilation, automatic tube compensation, pressure support ventilation, proportional assist ventilation, neurally adjusted ventilatory assist

## Methods of liberation from mechanical ventilation: Which one is best?

As an essential life-saving intervention, mechanical ventilation is also associated with complications which result to higher medical costs and mortality (1, 2). Therefore, it was essential to liberate patients from mechanical ventilation efficacy and safety for the shortest possible duration. Despite many studies comparing the safety and effectiveness of different methods for weaning have been published, many controversial questions remain concerning the best method for this process. Given that studies to date have not investigated the comparative of all available modes of weaning simultaneously, a network meta-analysis may help evaluate the relative effectiveness between all modes from both direct and mixed-treatment comparisons (3).

There were two network meta-analysis focuses on the best weaning methods published in this research topic of acute respiratory distress syndrome and mechanical ventilation. Although the study selection criteria were not identical, 12 randomized controlled trials (RCTs) were overlapped in the two studies. The study by Yi et al. including 24 RCTs showed that automatic tube compensation (ATC) obtained superior weaning success compared to T-piece and pressure support ventilation (PSV). Another study by Jhou et al. including 39 RCTs compared the efficacy among 7 modes of weaning and provided evidence that proportional assist ventilation (PAV) has a high probability of being the most effective ventilation mode regarding a higher rate of weaning success, a lower reintubation, and mortality rate. The features of pivotal clinical trials included in the meta-analysis are presented in the Table 1 (4–10). The reliability of these findings should be interpreted cautiously for several reasons. First, these findings were generated from single-center trials with limited sample size. Second, the difficulty of weaning (simple weaning, difficult weaning, and prolonged weaning) and duration of mechanical ventilation vary across studies, which has potential influence on the results of weaning outcome and may introduce a potential bias. Third, the variety of sedation and ventilation setting prior to or during liberation process also impact the clinical efficacy and introduce a potential bias. Further multicenter studies considering different clinical vignettes and respiratory physiology patterns are warranted to gain full insight into the real role of various weaning methods.

**Table 1:** Features of pivotal clinical studies.

**References**	**Population**	**Interventions**		**Strength**	**Weakness**
		**Control group**	**Experimental group**		
Esteban et al. (4)	484 ICU patients	T-piece; T-piece for a maximum of 2 h	PSV Pressure support of 7 cm H_2_O and PEEP ≤ 5 cm H_2_O	• Multicenter randomized design • The result supported SBT with pressure support or T-tube are suitable methods for extubation	• patients received longer mechanical ventilation before the SBT • the imbalances of patients after randomization
Chittawatanarat et al. (5)	520 SICU postoperative patients	T-piece, with an oxygenation setting of 10–15 L/min	PSV: inspiratory pressure 5–7 cm H_2_O, PEEP 5 cm H_2_O	• The randomized control trial • Surgical patient	• unblinded study design • prolong ventilator use
Subirà et al. (6)	1,153 ICU patients	T–piece for 2 h	PSV: 30-min with pressure support 8 cm H_2_O and zero PEEP	• Multicenter randomized design • Large sample size • The results supported the use of a shorter, less demanding strategy of 30 min of pressure support ventilation for SBT	• unblinded study design • non-protocolized extubation strategies
Xirouchaki et al. (7)	208 ICU patients	PAV+: the initial percentage of assist was set to 60–80%a/	VCV/PCV to PSV: PSV: the inspiratory pressure was set to 20–25 cm H_2_O (including PEEP_E_)	• The result supports PAV+ may be used as a mode of support in critically ill patients	• single center • lack information on weaning time • unblinded study design
Botha et al. (8)	50 ICU patients	PAV+: 70% support and weaned to 30% support by decrements of 10% as tolerated	PSV: Start with pressure support level required and weaned to 10 cm H_2_O as tolerated	• Appropriate number of patients enrolled • First RCT with PAV+ with a study period longer than 48 h • Well study protocol	• poor generalizability
Cohen et al. (9)	99 ICU patients	PSV to ATC; ATC: ventilator circuit with flow-triggering (2 L/min) and CPAP of 5 cm H_2_O, with inspiratory ATC set at 100%	PSV to CPAP; CPAP: ventilator circuit with flow triggering (2 L/min) and CPAP of 5 cm H_2_O	• The largest single-center study to assess the use of commercially available ATC	• No formally assess the technical performance of ATC
Taniguchi et al. (10)	70 ICU patients	SmartCare	PSV; Pressure 5–7 cm H_2_O and PEEP 5 cm H_2_O	• The result confirmed the efficiency of respiratory physiotherapy–driven weaning protocol	• small sample size • poor generalizability • the effectiveness of SmartCare™ performance during weaning phase of invasive MV

ICU, intensive care unit; PSV, pressure support ventilation; SICU, surgical intensive care unit, PAV, proportional assist ventilation; VCV, voulme control ventilation; PCV, pressure control ventilation; ATC, automatic tube compensation; CPAP, continuous positive airway pressure; MV, mechanical ventialtion.

Nonetheless, these findings promote pondering deeply over the criteria for the ideal method of ventilator liberation. PSV is the most commonly used mode of weaning in recent decades. In PSV mode, the PS can decrease the work of breathing imposed by the endotracheal tube (11). Short duration of PSV with a low level of assistance was also recommended by the most recent guidelines performed as initial spontaneous breathing trial rather than T-piece or CPAP (12). The network meta-analysis also showed that PSV increased the rate of weaning success when compared with T-piece. However, PSV can only provide a constant positive pressure which may not match the patient's respiratory demand. Of note, Yi et al. found that PAV was superior to PSV regarding weaning success, and Jhou et al. found that ATC was also superior to PSV. A sizeable effect with patient-ventilator asynchrony and over-assistance during PSV weaning might be a possible explanation (13). PAV, which delivered positive pressure ventilation in proportion to instantaneous inspiratory effort, was associated with less patient ventilator asynchrony and lower risk of over-assistance (14). Nevertheless, PAV is relatively complex; indeed, the settings need knowing or estimating the patient's compliance and resistance (15). ATC, which delivered dynamic positive pressure automatically to compensate for the resistance of artificial airway, can improve synchronization between patient and ventilator, and avoided over-assistance (16, 17). However, ATC cannot increase lung ventilation heterogeneity as compared to low PS and PEEP (18). Nonetheless, unloading the respiratory muscle without over-assistance and better patient-ventilator interaction might be essential to the ideal method of weaning.

Neurally adjusted ventilatory assist (NAVA) mode uses the electrical activity of the diaphragm to control the ventilator and delivers pressure support in proportion to patients' neural effort. It has been demonstrated that NAVA improved patient-ventilator interaction and reduced inappropriate ventilator assist when compared with PSV (19, 20). Despite limited real-world experience, NAVA might be ideally suitable for the weaning process. Several studies have shown that NAVA improves the weaning outcome when compared with PSV, especially for patients difficult to wean (13, 21, 22). However, RCTs, comparing the safety and effectiveness between NAVA and other weaning modes, such as PAV and ATC, are absent.

Although, there is still controversy about the best method of liberation from mechanical ventilation, new mode in line with respiratory physiology might be a light at the end of the tunnel.

## Author contributions

LL wrote the original version and revised the Editorial.

## Funding

This study was supported by Key Research and Development Plan of Jiangsu Province (BE2020786) and 333 High Level Talents Training Project in the sixth phase in Jiangsu.

## Conflict of interest

The author declares that the research was conducted in the absence of any commercial or financial relationships that could be construed as a potential conflict of interest.

## Publisher's note

All claims expressed in this article are solely those of the authors and do not necessarily represent those of their affiliated organizations, or those of the publisher, the editors and the reviewers. Any product that may be evaluated in this article, or claim that may be made by its manufacturer, is not guaranteed or endorsed by the publisher.
